# Is there a distinction between malaria treatment and intermittent preventive treatment? Insights from a cross-sectional study of anti-malarial drug use among Ugandan pregnant women

**DOI:** 10.1186/s12936-015-0702-7

**Published:** 2015-05-03

**Authors:** Charles O Odongo, Kuteesa R Bisaso, Freddy Kitutu, Celestino Obua, Josaphat Byamugisha

**Affiliations:** Department of Pharmacology and Therapeutics, School of Biomedical Sciences, College of Health Sciences, Makerere University, Kampala, Uganda; Department of Pharmacology and Therapeutics, Faculty of Medicine, Gulu University, Gulu, Uganda; Department of Pharmacy, School of Health Sciences, College of Health Sciences, Makerere University, Kampala, Uganda; Mbarara University of Science and Technology, Mbarara, Uganda; Department of Obstetrics and Gynaecology, School of Medicine, College of Health Sciences, Makerere University, Kampala, Uganda

**Keywords:** Malaria treatment, Intermittent preventive treatment, Sulphadoxine-pyrimethamine, Pregnant women, Uganda

## Abstract

**Background:**

In Uganda, treatment of clinical malaria and intermittent preventive treatment with sulphadoxine-pyrimethamine (SP) are common during pregnancy. As a result, both formal and informal reports from antenatal sources suggest possible misuse of SP for malaria treatment among pregnant women. The objective of this study was to investigate anti-malarial drug use patterns among women who had recently suffered malaria illness before and during pregnancy.

**Methods:**

A cross-sectional study in which a structured questionnaire (interviewer-administered) was used to collect data from pregnant women attending an urban antenatal clinic. Details of medicines used to treat malaria episodes suffered before and during pregnancy were captured. A first order Markov probability model was used to estimate probabilities of transitioning between treatment choices made before and during pregnancy. Logistic regression was used to explore whether demographic and obstetric characteristics were associated with transition patterns.

**Results:**

Seven hundred women were interviewed among whom 428 had suffered malaria in both instances. Three hundred thirty of these could recall the medicines used in both instances. Women who used ACT/QNN (correct choice) before pregnancy had higher probabilities of transitioning to SP than staying on ACT/QNN during pregnancy (0.463 versus 0.451). Access of medicines from private outlets (clinics and pharmacies) were more than nine times predictive of receiving correct medicines (p=0.035 and p=0.039 respectively). Access of medicines from clinics was 5.9 times protective against receiving SP for malaria treatment (p=0.033). Among those who used SP before pregnancy, there was a 0.75 probability of staying on it during pregnancy. None of the factors explored could explain this observation.

**Conclusion:**

Use of SP for malaria treatment is common during pregnancy. This may be contributing to adverse pregnancy outcomes. Antenatal care providers should endeavour to emphasize the distinction between treatment and prevention of malaria during pregnancy.

**Electronic supplementary material:**

The online version of this article (doi:10.1186/s12936-015-0702-7) contains supplementary material, which is available to authorized users.

## Background

Among adult populations in sub-Saharan Africa, pregnant women are disproportionately affected by malaria. The World Health Organization (WHO) recommends a combination of measures to mitigate the adverse impact of malaria during pregnancy. These include; use of insecticide-treated bed nets, early case detection and treatment (with an effective anti-malarial medicine) as well as intermittent preventive treatment during pregnancy (IPTp) [1]. Each year, more than thirty million pregnancies at risk of malaria occur in Africa [2] and of these, Uganda accounts for a considerable proportion mainly for two reasons; First, Ugandan women have the third highest fertility rate in the world [3]. Secondly, malaria is endemic in 95% of Uganda’s geographical territory which include places known to have some of the highest transmission rates ever recorded [4]. While recent efforts to control malaria in Uganda have registered remarkable gains, this dual burden most likely predisposes Ugandan women to high tendencies to consume anti-malarial medicines.

Whereas sulphadoxine-pyrimethamine (SP) remains the most appropriate drug for IPTp, the WHO recommends artemisinin-based combination therapy (ACT) as first-line treatment for uncomplicated malaria in the second and third trimesters of gestation [1]. ACT is however contraindicated in the first trimester of gestation [5] owing to limited safety data on artemisinins in early pregnancy. Instead, oral quinine (QNN) plus clindamycin given for seven days, is the recommended first-line treatment for malaria in this period [1]. The treatment policy for malaria during pregnancy in Uganda is largely similar to that of the WHO [6]. However, effective treatment in the first trimester is often challenging as clindamycin may not be affordable or readily available. This has led to common use of QNN monotherapy with poor adherence due to the relatively long treatment course (7 days) coupled with numerous side effects [7]. Furthermore, contrary to current guidelines, presumptive treatment of malaria remains high in many resource-limited settings [8,9]. This practice has persisted due to continuous challenges with access and delivery of reliable diagnostic services [10]. Needless to say, presumptive treatment leads to wasteful consumption of anti-malarial medicines [9,11] and significantly delays treatment of other febrile illnesses [11,12] and as a result, may portend adverse consequences.

Whereas antenatal care (ANC) clinics have the potential to effectively serve as delivery platforms for malaria control interventions in pregnancy, access to quality ANC remains a challenge in many African settings [13-15]. The Uganda Reproductive Health Policy recommends a minimum of four ANC visits in which a complete package can be delivered [16]. A woman with a normal pregnancy is expected to make at least one visit in each of the first and second trimesters and two visits in the third trimester. During these visits, women are supposed to receive malaria control interventions such as insecticide-treated bed nets, SP- IPTp, and most importantly, knowledge and awareness information on malaria prevention and its treatment. However, both formal [17,18] and informal reports from pregnant women in recent times continue to suggest SP misuse for malaria treatment. The broad objective of this study was to investigate the knowledge and practice gaps associated with malaria treatment among Ugandan pregnant women. This report presents insights from anti-malarial drug use patterns among a population of Ugandan women attending an urban antenatal clinic.

## Methods

### Study design

This was a cross-sectional study that employed quantitative methods of data collection and analysis. Exit interviews were used to collect data from pregnant women attending ANC at Mulago Hospital. Data for this study was obtained as part of a larger cross-sectional study on malaria, IPTp and anti-malarial drug use among Ugandan women, some of which was recently published [19].

### Study site and study population

Mulago Hospital is a 1,500 bed complex located in Kampala, the capital and commercial centre of Uganda. This is a public facility that serves as Uganda’s national referral and teaching hospital. In addition to offering a wide range of specialized care, the hospital runs specialized outpatient clinics on week days. Most outpatients come from the urban and peri-urban communities of Kampala and the neighbouring districts of Mukono, Wakiso and Mpigi. The majority of outpatients visiting the hospital tend to come from low and middle income families who prefer to utilize the hospital’s free services. The hospital runs two ANC clinics located at Old- and New Mulago complexes. The two clinics offer general (routine) and referral ANC services respectively. The study was conducted at the Old Mulago clinic because it was the first point of call for all pregnant women seeking ANC at the hospital.

### Research instrument

A structured questionnaire developed from our objectives based on the conceptual model proposed by Ribera *et al.* [20], was used to collect data from participants. Items covered addressing the present objective included a recall of any malaria episode prior to the current pregnancy including the treatment thereof. In addition, if the women had ever suffered from malaria during pregnancy (current or recent pregnancy), the names and sources of medicines used were also recorded. If SP was mentioned, care was taken to distinguish between its use for treatment of symptomatic malaria and its use as IPTp (the latter is standard care for all women attending ANC in Uganda [6]). Additional data collected included participant’s age, highest education level attained, parity (gestational age ≥ 28 weeks) and number of ANC visits in their most recent pregnancy. The complete questionnaire is available as Additional file 1. Prior to data collection, the questionnaire was pre-tested on fifty women from the same clinic. This allowed the research team to cater for all possible responses as well as standardize questions so as to ensure internal validity. Detailed data collection methods have been recently published [19].

### Sample size determination and selection criteria

Using the survey formula of Cochran [21], a minimum sample size of 303 participants was required based on the following parameter estimates; a 5% level of precision, a standard normal deviate (Zα) of 1.96 (for a confidence level of 95%) and a 27% variability level for women who develop malaria during pregnancy based on a recent study conducted in a similar setting [22]. However, in order to qualify for the present analysis, participants had to have experienced malaria before as well as during pregnancy. Therefore, women were consecutively approached without exclusion, and invited to participate in the study. This strategy allowed for random selection and attainment of the desired number of participants.

### Recruitment and data collection

Recruitment and data collection for the entire study was done over three weeks in August 2013. Pregnant women leaving the consultation rooms were consecutively approached and invited to participate. Three female students of nursing were trained and assisted in this exercise. On each clinic day, data collection begun at 09:00 and ended at 13:00 hours. Each research assistant administered the questionnaire to an average of 15 pregnant women per day, with each interview lasting between ten to fifteen minutes. The interviewers were free to ask additional specific questions so as to satisfy themselves that anti-malarial medicines were not being confused with other routine ANC medications. Communication was either in English or a local vernacular language (whichever was convenient for individual women). Whilst in the clinic, all completed questionnaires were scrutinized by the investigators so as to ensure correctness and completeness of records before they were filed.

### Ethical considerations

The study protocol received ethical approval from the hospital’s research and ethics committee (Approval Ref. No. MREC 397) as well as the Uganda National Council for Science and Technology (UNCST). Oral consent was obtained from each participant prior to interviews. Convenience and confidentiality were observed by interviewing each participant in privacy within the clinic premises.

### Data management and statistical analysis

Data was coded, double entered and validated in *EpiData*® software [23] from where it was exported to R statistical computing software for analysis [24]. In order to analyse drug use patterns for malaria treatment before and during pregnancy, a Markov probability model was proposed. Three main transition states were identified and coded as follows:

- **State 1-ACT/QNN:** Either of these medicines was considered a correct treatment hence the two were combined (since we were unable to determine the actual gestational age at which malaria episode occurred).
- **State 2-SP:** This was considered the misguided choice (arising out of misconception about its indication, possibly due to confusion with its common use as IPTp).
- **State 3-OTHERS:** Consisting of all other incorrect medicines that were mentioned, including chloroquine, antibiotics and herbal remedies.

**Figure 1:**
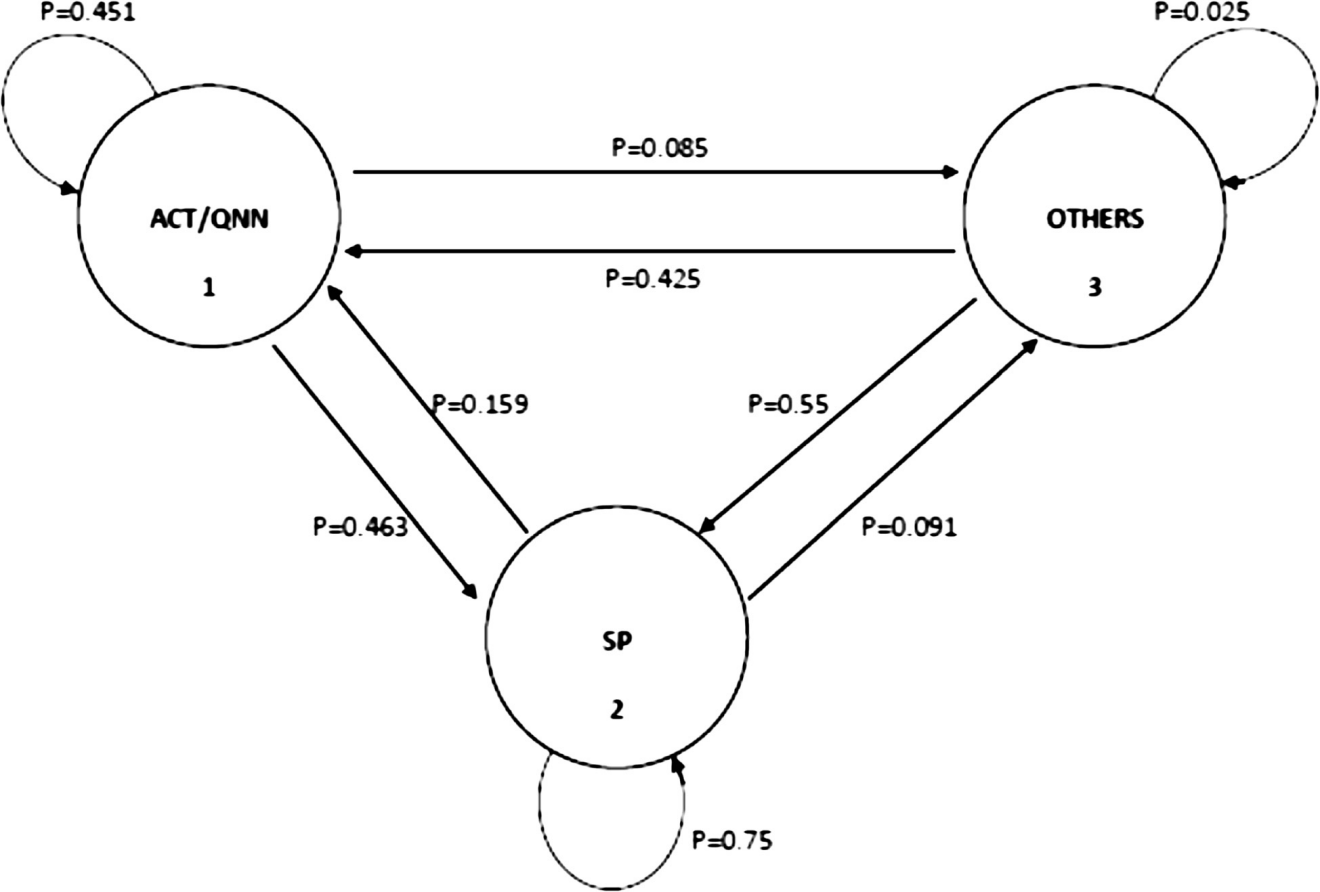
Markov probability model showing treatment choice transitions before and during pregnancy.

In order to construct the probability model presented in Figure 1, two assumptions were made namely; i) a constant time interval for each woman, between malaria episodes suffered before and during pregnancy, and ii) each medicine was considered used in its rightful dose and duration at all times. Furthermore, Anderson and Goodman’s maximum likelihood ratio criterion [25] was used to test for the Markov property in the data prior to modelling. The null hypothesis was independence of medicine choice before and during pregnancy, while the alternative hypothesis was that choices exhibited partial dependence. With a chi square value of 51 against 4 degrees of freedom, the null hypothesis was rejected (*p* < 0.000). Thus, the data was analysed using a first order Markov model. Transition probabilities were estimated by a simple non-parametric count method. Variables such as ‘source of medicine’ as well as demographic and obstetric characteristics were investigated as possible predictors of transitioning between states. Multivariate logistic regression analysis of covariate dependence [26] was performed on six transition scenarios which were of interest (see Figure 1) namely;

i. Use of ACT/QNN both before and during pregnancy (1 → 1),
ii. Use of ACT/QNN when not pregnant, then transitioning to SP when pregnant (1 → 2),
iii. Use of SP both before pregnancy and during pregnancy (2 → 2),
iv. Use of SP when not pregnant and transitioning to ACT/QNN when pregnant (2 → 1),
v. Use of OTHERS when not pregnant and transitioning to SP when pregnant (3 → 2)
vi. Use of OTHERS when not pregnant then ACT/QNN when pregnant (3 → 1).

Odds ratios were computed for each transition with statistical significance set at p ≤ 0.05 at the 95% confidence level.

## Results

### Demographic characteristics of respondents

Seven hundred respondents were interviewed in order to identify 428 women who had suffered malaria before as well as during pregnancy. Three hundred and thirty of these could correctly recall the medicines used to treat the two malaria episodes and thus constituted the sample for the present analysis. Median age in this group was 25 years (min. 19, max. 41). Most had completed primary (36.1%) or basic secondary (25.2%) education. Only 24.5% had attained advanced secondary education or other higher qualifications while 14.2% had never attended (or failed to complete) primary education. First- and second-time mothers respectively, comprised 16.4% and 48.4% of the sample, while women who had experienced higher number of pregnancies comprised 35.1% of the sample. Excluding first-time mothers, 82.1% of women had attended between 3–7 ANC visits during their most recent pregnancy. Only 13.6% had attended 1–2 times while 4.3% never attended ANC in their most recent pregnancy. When asked to mention the sources of medicines used for treatment of malaria episodes suffered during pregnancy, 50.0% (n=165) reported accessing medicines from a private clinic while 29.1% accessed from a hospital. Other sources of medicines were public health centres 8.5%, private pharmacies 8.8%, and community drug shops 3.6%.

### State occupancy and transition probabilities for medicines used before and during pregnancy

Overall, ACT/QNN were the most commonly used treatment before pregnancy while SP was most commonly used during pregnancy. This was true regardless of the medicines used before pregnancy. Table 1 presents state prevalence and probability estimates for use of anti-malarial medicines before, then during pregnancy. Figure 1 presents the transition probability estimates (from Table 1) in form of a Markov model diagram. Among women who used ACT/QNN before pregnancy, there was a higher probability of switching to SP (p=0.463) than remaining on ACT/QNN (p=0.451) during pregnancy. Among those who used SP before pregnancy, there was a higher probability of remaining on SP (p=0.75) than switching to ACT/QNN (p=0.159) during pregnancy. Among those who used ‘OTHERS’ before pregnancy, there was a higher probability of switching to SP (p=0.55) than switching to ACT/QNN (p=0.425).

**Table 1:** **State prevalence and transition probability matrix for anti-malarial treatments used before and during pregnancy**

	**State prevalences (%) n=330**		**Transition probabilities**		
	**Before pregnancy**	**During pregnancy**	**→ ACT/QNN**	**→ Others**	**→ SP**
**ACT/QNN →**	74.5	41.9	0.451	0.085	0.463
**Others →**	12.1	7.9	0.425	0.025	0.550
**SP →**	13.3	51.2	0.159	0.091	0.750

### Factors determining treatment choice transitions during pregnancy

Of the six transition scenarios for which multivariate analysis was performed, significant findings were seen in only two scenarios; 1: use of ACT/QNN in both instances, before and during pregnancy (1 → 1), and 2: use of ACT/QNN before pregnancy then switching to SP during pregnancy (1 → 2). Of all predictor variables investigated, only ‘source of medicine’ significantly predicted transitions in the two scenarios. Women who used ACT/QNN prior to pregnancy were 9.8 and 11.4 times likely to stay on it if the medicine was accessed from a private clinic or private pharmacy respectively (Table 2). Among women who used ACT/QNN before pregnancy, accessing medicines from a private clinic was 5.9 times protective against switching to SP during pregnancy. A similar trend was noticed with all other sources of medicines although they did not reach statistical significance (see Table 2).

**Table 2:** **Multivariate regression analysis of predictors of transitioning between malaria treatments received before and during pregnancy**

**Variables**	**Log odds**	**Std. error**	OR* (C.I.)	**1/OR** ^**†**^	**p-value**
**Transition scenario ACT/QNN before and during pregnancy (1** → **1)**					
Constant	−1.298	1.461	0.273		0.374
Age	−0.032	0.043	0.969 (0.89–1.05)	1.03	0.456
Education level	0.049	0.119	1.05 (0.83–1.34)		0.678
Parity	−0.107	0.135	0.899 (0.69–1.68)	1.11	0.423
No. of ANC visits	0.011	0.098	1.011 (0.83–1.23)		0.913
Private clinic	2.286	1.085	9.84 (1.69–18.6)		**0.035**
Pharmacy	2.429	1.178	11.35 (1.53–23.8)		**0.039**
Public Health center	1.195	1.170	3.3 (0.44–6.8)		0.307
Hospital	2.009	1.098	7.46 (1.24–14.3)		0.067
**Transition scenario ACT/QNN to SP before and during pregnancy (1** → **2)**					
Constant	0.267	1.267	1.31 (0.11–18.13)		0.833
Age	0.021	0.043	1.02 (0.94–1.11)		0.626
Education level	−0.038	0.119	1.04 (0.76–1.22)		0.754
Parity	0.169	0.134	1.18 (0.91–1.55)		0.208
No. of ANC visits	0.089	0.098	1.09 (0.90–1.33)		0.359
Private clinic	−1.776	0.834	0.169 (0.02–0.75)	5.9	**0.033**
Pharmacy	−1.684	0.954	0.186 (0.02–1.08)	5.4	0.078
Public Health center	−1.504	0.915	0.222 (0.03–1.19)	4.5	0.10
Hospital	−1.506	0.850	0.221 (0.03–1.02)	4.4	0.076

*OR: odds ratio, ^**†**^inverse operation applied to OR estimates less than 1 only.

boldface figures indicate statistical significance at p less than 0.05 at the 95% confidence level.

## Discussion

This study used a transition probability model to show changes in malaria treatment patterns among pregnant women in Uganda. Women were more likely to use SP than ACT/QNN, to treat reported episodes of malaria during pregnancy. The prevalence and probability patterns revealed here show that women (or their treatment providers) consciously chose to use SP for malaria treatment, highlighting possible existence of confusion about its role (indication) during pregnancy. High levels of awareness about SP as an anti-malarial drug coupled with low knowledge about its indication during pregnancy has been previously reported in this study population [19]. This critical knowledge deficit may be attributed to the low education levels considering that more than half of the women had received limited (primary) or no formal education. It is, therefore, probable that they did not easily comprehend the distinction between treatment and prevention of malaria and the drugs thereof. Because patients in developing countries most often trust and follow malaria treatment advice offered by health workers [27], these results may be a reflection of health workers’ lack of focussed malaria treatment information. Alternatively, health workers may be delivering this information in a manner that is inappropriate to recipients’ needs, given their low literacy and low knowledge base, thus leaving them ‘half-baked’.

Many previous studies have highlighted the high rates of self-medication and informal treatment-seeking behaviour in Uganda [27-30]. In light of these considerations, the present findings raise two disturbing concerns; first, unless SP-IPTp is administered under the strict supervision of a health worker, women who do not perceive themselves as ill with malaria (as at routine ANC visits), are unlikely to take SP-IPTp by themselves. As such, questions as to whether pregnant women comply with unsupervised intake (of SP-IPTp) remain valid [19]. Secondly, SP lacks efficacy against clinical malaria. Its use for malaria treatment may, therefore, be associated with high risks of treatment failure and the consequences thereof. These concerns may not be far-fetched considering the high awareness about SP, its inexpensiveness and wide availability in many drug outlets within Uganda, often with inadequate regulatory oversight. It is, therefore, possible that consequences of inappropriate use of SP significantly contribute to the high incidences of poor pregnancy outcomes in Uganda. In a recent study from Mukono district (Uganda), Mbonye et al. showed a high prevalence of self-medication with SP among cotrimoxazole-taking HIV positive pregnant women presenting with a febrile illness [17]. Clearly, such practices underscore the misconception about SP as a treatment for malaria during pregnancy.

As with many studies from Uganda and elsewhere [29-33], private drug outlets were an important source of anti-malarial medicines in this study. This may be due to their common location within residential areas where they act as points of first call for remedies to acute illness. Interestingly in this study, access of anti-malarial medicines through private drug outlets appeared to guard against inappropriate use of SP. This finding is contrary to past studies that have suggested that treatment decisions among private or informal health care providers were often irrational, driven more by profit motives than clients’ interests [34]. Despite the fact that we did not determine actual prescribers of the medicines reported in this study, the possibility of self-treatment cannot be under-estimated since the purchase of medicines without a prescription is not uncommon in this society as cited above. Evidence from previous studies suggests that both patients and prescribers alike, contribute to the culture of irrational drug-use. In the study from Mukono cited above, 10.9% of parasite-confirmed and 48.6% of parasite-negative pregnant women received SP as treatment from health centre staff [17]. Therefore, both parties may be responsible for the irrational pattern of SP use observed in this study.

The findings from this study may have two important limitations, namely respondents’ ability to distinguish malaria from other illnesses as well as recall bias to do with event details. Whereas nearly all data collected in this study reflected events that happened more than one month earlier, malaria is not just an event in this society, rather, it is a very common experience that respondents easily relate with. As such, many adults, especially household heads (as in this case) have vivid ideas on its symptoms and popular remedies. Therefore, accurate memory about the details around malaria episodes were likely to be high. Furthermore, only respondents who could clearly identify anti-malarial medicines by name or description were included in this analysis. Ninety-eight potential respondents who failed to clearly articulate names or descriptions of the medicines used in both states were excluded. Thus, the above limitations were significantly minimized within the limits of the cross-sectional study design and the research tools employed.

## Conclusion

This study highlights the inappropriate use of SP for malaria treatment among pregnant women in Uganda. In order to address this problem and improve malaria treatment during pregnancy, health workers should at all times endeavour to provide appropriate information on malaria treatment. Additional emphasis should be placed on the distinction between prevention and treatment of clinical malaria during pregnancy.

## Additional file

Additional file 1: Knowledge and experiences with malaria and sulphadoxine-pyrimethamine (Fansidar) among pregnant women at Mulago referral hospital, Kampala. This is a three-part, twenty-item, research instrument (questionnaire) used to collect data on knowledge and experiences with malaria and anti-malarial drug use among Ugandan pregnant women.

## Abbreviations

ACT: Artemisinin combination therapy
ANC: Antenatal care
IPTp: Intermittent preventive treatment of malaria during pregnancy
QNN: Quinine
SP: Sulphadoxine-pyrimethamine
WHO: World Health Organization
